# BulkVis: a graphical viewer for Oxford nanopore bulk FAST5 files

**DOI:** 10.1093/bioinformatics/bty841

**Published:** 2018-11-20

**Authors:** Alexander Payne, Nadine Holmes, Vardhman Rakyan, Matthew Loose

**Affiliations:** 1 School of Life Sciences, University of Nottingham, Nottingham, UK; 2 DeepSeq, School of Life Sciences, University of Nottingham, Nottingham, UK; 3 The Blizard Institute, Barts and The London School of Medicine and Dentistry and Centre for Genomic Health, LSI, Queen Mary University of London, London, UK

## Abstract

**Motivation:**

The Oxford Nanopore Technologies (ONT) MinION is used for sequencing a wide variety of sample types with diverse methods of sample extraction. Nanopore sequencers output FAST5 files containing signal data subsequently base called to FASTQ format. Optionally, ONT devices can collect data from all sequencing channels simultaneously in a bulk FAST5 file enabling inspection of signal in any channel at any point. We sought to visualize this signal to inspect challenging or difficult to sequence samples.

**Results:**

The BulkVis tool can load a bulk FAST5 file and overlays MinKNOW (the software that controls ONT sequencers) classifications on the signal trace and can show mappings to a reference. Users can navigate to a channel and time or, given a FASTQ header from a read, jump to its specific position. BulkVis can export regions as Nanopore base caller compatible reads. Using BulkVis, we find long reads can be incorrectly divided by MinKNOW resulting in single DNA molecules being split into two or more reads. The longest seen to date is 2 272 580 bases in length and reported in eleven consecutive reads. We provide helper scripts that identify and reconstruct split reads given a sequencing summary file and alignment to a reference. We note that incorrect read splitting appears to vary according to input sample type and is more common in ’ultra-long’ read preparations.

**Availability and implementation:**

The software is available freely under an MIT license at https://github.com/LooseLab/bulkvis.

**Supplementary information:**

Supplementary data are available at *Bioinformatics* online.

## 1 Introduction

Oxford Nanopore Technologies (ONT) range of sequencing platforms (MinION, GridION and PromethION) utilize biological nanopores, embedded in synthetic membranes, to sequence individual single-stranded molecules of DNA (Jain *et al.*, 2015). As DNA passes through the nanopore it creates sequence specific disruptions in current flow (Ip *et al.*, 2015). The resultant reads are written to disk as soon as the DNA has translocated the pore; uniquely enabling rapid analysis of sequence data ideal for both field and clinical work (Euskirchen *et al.*, 2017; Quick *et al.*, 2016). The software controlling sequencing (MinKNOW) does this by monitoring the flow cell in real time to determine if the signal observed from each channel represents DNA. MinKNOW processes the continuous data stream from the sequencer into individual read FAST5 files containing raw signal data that are subsequently base called to reveal the sequence.The sequence of the DNA can even be analysed while the DNA is in the pore, enabling approaches such as ‘Read Until’ where specific molecules can be dynamically rejected according to user customisable parameters (Loose *et al.*, 2016).

Partitioning the real-time data stream into reads results in information loss about the current state before and after an individual read. To better understand these events and view the effects of user intervention on sequencing when developing methods for read until or using difficult samples, we wished to visualize the entire data stream from the MinION device. ONT provide an optional bulk FAST5 file format to capture the entire data stream from every channel on the sequencing device (see http://bulkvis.readthedocs.io for guidance on how to collect a bulk FAST5 file). This file includes raw signal and metadata for every channel including MinKNOW classifications (see Supplementary Table S1). To visualize bulk FAST5 files, we developed BulkVis using the bokeh visualization package (https://bokeh.pydata.org). BulkVis annotates signal features based on the metadata and optionally mappings from a PAF file. These annotations provide a simple method to relate base called reads back to the channel and time in the data stream from which they originate and visualize their genomic location.

Whilst developing BulkVis, we observed examples of reads incorrectly segmented by MinKNOW leading to a reduction in the read lengths reported. This incorrect splitting of reads appears to correlate with read lengths such that ultra-long reads are more likely to be affected. In some cases there is no apparent reason for the read to have been split, but in many others we observe examples of reads that exhibit unusual signal patterns prior to the incorrect split.

## 2 Results

**Fig. 1.**
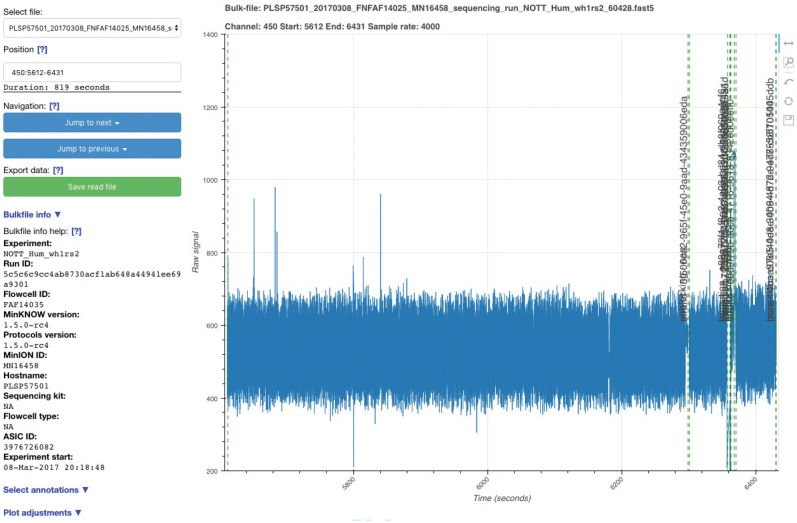
Screenshot of the BulkVis application running. The vertical dashed lines indicate different annotations overlaid by MinKNOW on the signal trace in real time. The left panel provides configuration and navigation options for the selected bulk FAST5 file

**Fig. 2.**
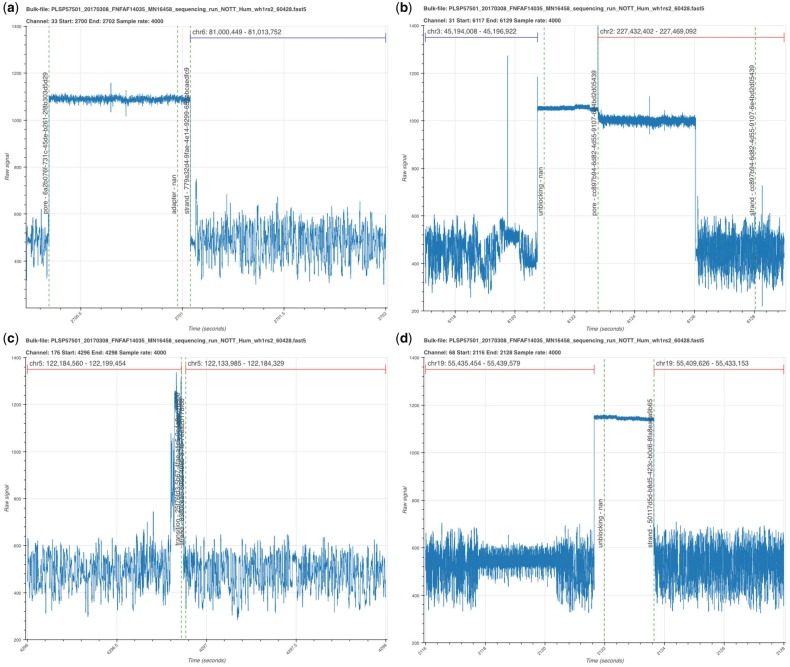
Illustrative segments from a bulk FAST5 file visualized with BulkVis. (**a**) The start of a read mapping to chromosome 6. Open channel ‘pore’, followed by an ‘adapter’, and ‘strand’ as annotated by MinKNOW. (**b**) Read ending with an ‘unblock’ followed by ‘pore’ and then a new read. (**c**) Adjacent reads from a channel separated by unusual current patterns.These two reads are reported as distinct molecules by MinKNOW, they map consecutively to the reference. (**d**) Two adjacent reads separated by an ‘unblock’ signal. The unblock does not successfully remove the DNA. Instead the read continues to sequence again mapping adjacently to the reference

BulkVis scans a folder containing bulk FAST5 files at startup. An individual file is selected and specific channels plotted (Fig. 1). Basic metadata are displayed to the user. To navigate coordinates are input in the format channel: start_time-end_time. Alternatively pasting a FASTQ read header from a base called read will jump to its channel, time and raw signal from the bulk FAST5 file. Files can also be navigated by jumping to the next or previous instance of a specific annotation (Supplementary Table S1). Annotations are overlaid on the signal plot as vertical dashed lines, labelled with the type and associated ID if available (Fig. 1); mappings, generated by gen_bmf.py, are overlaid horizontally above the signal, with blue/red indicating forward/reverse mappings, respectively (Fig. 2). Raw signal data are proportionally smoothed to aid rapid visualization. BulkVis allows export of the signal section being viewed to a read FAST5 file compatible with Nanopore base callers. To avoid confusion with MinKNOW derived reads, BulkVis reads are custom named and include the channel number and the start and end index of the read segment recorded in samples. The read segment shown in Figure 1 results in a read FAST5 file named plsp57501_20170308_fnfaf14035_m n16458_sequencing_run_nott_hum_wh1rs2_60428_bulkvis-read_22448000-25724000_ch_450.FAST5. This region captures three single reads that when called as one read generates a 215 662 base sequence (Supplementary File Collection S1). The three individual reads base call with a combined length of 215 153 bases (Supplementary File Collection S1) and the single called read maps well to the original three (Supplementary Fig. S1a).

During library preparation, adapter sequences are added to DNA molecules such that every sequenced read should begin with an adapter sequence. MinKNOW classifies sequences in real time, usually labeling read starts with the annotation ‘adapter’. A channel without DNA in a pore will be labelled ‘pore’. Typically then adapter sequences should be detected (labelled ‘adapter’) followed by the signal derived from the read itself (‘strand’) (Fig. 2a). BulkVis was developed in part to observe the effects of unblocking (the reversal of voltage across a specific channel to eject material from the pore) on DNA sequence in a nanopore. Unblocking is used in two ways; firstly the sequencer detects and removes blockages in the pore and, secondly, for the rejection of unwanted DNA in selective sequencing or ‘Read Until’ (Loose *et al.*, 2016). To observe the effect of an unblock (Fig. 2b) on a channel immediately after the read has been ejected users must analyse a bulk FAST5 file. Alternatively reads can be inspected in order from an individual channel. For the data presented here, unblocks have a fixed duration of 2 seconds after which the channel should return to its normal state. ONT have released an updated version of unblock, termed ‘Progressive Unblock’ that gradually increases the duration of the flick time (MinKNOW 2.0 Stuart Reid Pers Comm.).

During recent efforts sequencing the human genome on a MinION (Jain *et al.*, 2018), a protocol to sequence ultra-long DNA molecules was generated by Quick (2018). We used BulkVis to investigate the signal from MinKNOW during one of these runs (ASIC ID 3976726082, Supplementary Note S1). We observed reads without the expected ‘pore’, ‘adapter’, ‘strand’ sequence. We found ‘strand’ sequences separated by either ‘above’ and/or ‘transition’ (Fig. 2c) or even ‘unblock’ (Fig. 2d) signals without any evidence of ‘pore’ or ‘adapter’ sequences present. This was surprising given that every sequenced read should begin with an adapter. We therefore closely examined reads before and after these unusual read split events. By looking at read mappings prior and post the events shown in Figure 2c and d, we determined the two sequences were derived from adjacent positions on the same chromosome (Table 1). These reads, sequenced one after another, were most likely derived from single molecules. The alternative explanation is the chance sequencing of two independent molecules that map adjacently on the human reference, one after another, through the same pore.

**Table 1.** Mapping data for events shown in Figure 2c and d

	Read ID	Chan	Read	Length	Chr	Start	End
2C	7ed4aafb-d058-481c-ad60-903fd8327240	176	943	10 275	5	122 184 560	122 199 454
	83d0cea6-69ad-406b-87fb-7eaa2b178f68			43 145		122 133 985	122 184 329
2D	c13c1e73-f7e0-4ae2-8cda-729f3b4dcb79	68	758	5068	19	55 435 454	55 439 579
	50117d5d-b8d5-423c-b0d6-8fa8eaea9b65			25 596		55 409 626	55 433 153

*Note*: Reads mapped to GRCh38 (minimap2 -x map-ont). Combined read length (2C) is 56 284 bases, mapping to a span of 65 469 bases. Combined read length (2D) is 30 664 bases, mapping to a span of 29 953 bases. All reads here map in reverse orientation.

Mapping all the reads (ASIC ID 3976726082) against the GRCh38 reference (Schneider *et al.*, 2017) and using read and channel numbers to sort by order through each channel we asked how many adjacent reads mapped to contiguous positions [whale_watch.py (Colloquially, Nanopore reads exceeding 1 Mb have been referred to as ‘whales’, with the species of whale determined by converting the length of a read in kb to a mass in kg, hence our script naming conventions.)]. About 2983 of 75 689 reads were incorrectly split with pairs of reads mapping adjacently to the reference. Stitching these reads together (using whale_merge.py) increased read length N50 from 98 876 to 103 925 bases. Mean read length of incorrectly split reads (55 190 bases) is higher than the entire dataset (23 717 bases). Re-examining previous ultra-long datasets revealed incorrect read splitting occurred 1-10% of the time (Supplementary Table S2). Incorrectly split reads had consistently higher mean read lengths than those which appear to be true single molecules. As such, these reads have significant effects on read N50 (up to 21 kb).

**Fig. 3.**
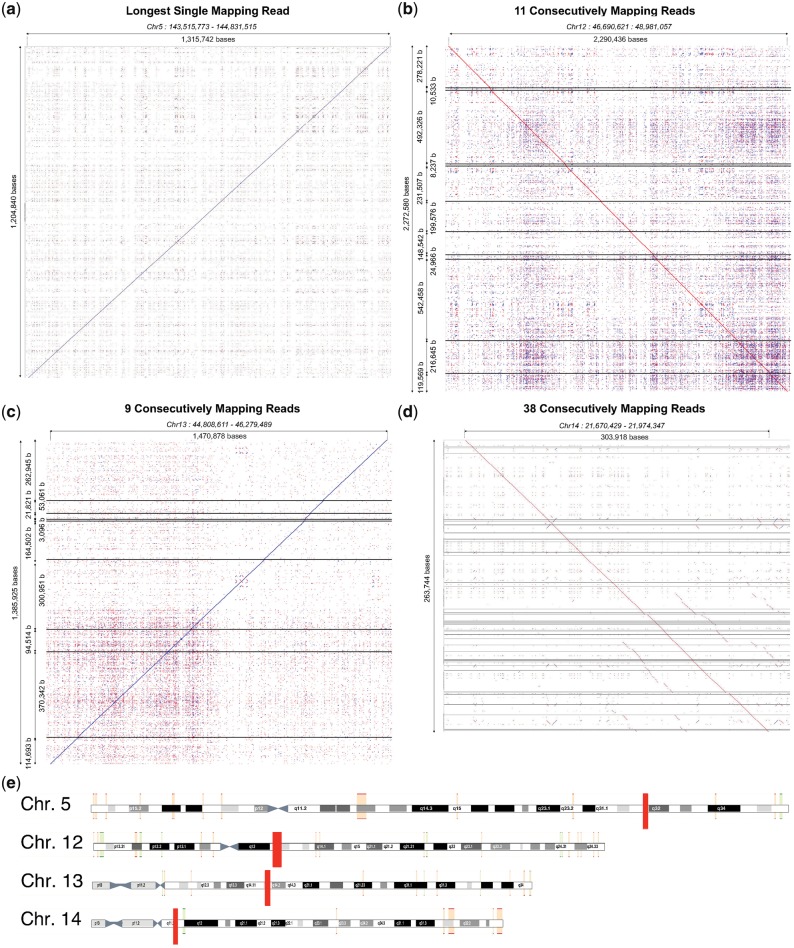
Read mappings. (**a**) Longest single read. (**b**) Longest fused read (>2 Mb), sequenced in 11 reads. (**c**) Longest fused read sequenced with a bulk FAST5 file. (**d**) Fused read comprising 38 individual sequences. (a–d) Reads mapped and visualized with last (-m 1) and last-dotplot (Kiełbasa *et al.*, 2011). Horizontal lines indicate breaks between individual reads. (**e**) Illustration of reads, shown as red rectangles, from A to D mapped against GRCh38 in ENSEMBL

We generated additional ultra-long reads from the same reference human genomic DNA sample using the RAD004 transposase kit for ultra-long reads (Jain *et al.*, 2018; Quick, 2018). This revealed more incorrectly split reads with up to 30% of reads in one run affected and increases in read N50 of up to 40 kb (data not shown). Differences between runs include the input DNA, the sequencing kit, other unknown variables within the flowcells and MinKNOW software itself. Within this dataset we found a single read of 1 204 840 bases that maps to 1 325 742 bases on chromosome 5 (Fig. 3a). Remarkably, we found a set of eleven reads that, when merged, were 2 272 580 bases in length. This merged read maps to a single location in the human genome spanning 2 290 436 bases (Supplementary Table S3, Fig. 3b, Supplementary File Collection S2). Unfortunately, we did not collect a bulk FAST5 file for this run. The next longest ‘fused’ read caught in a bulk FAST5 file was 1 385 925 bases in length, derived from nine individual reads (Supplementary Table S4, Fig. 3c, Supplementary Fig. S2). Using BulkVis we created a single read FAST5 file from the signal covering all these reads and base called it using albacore resulting in a read that maps in its entirety to a single location in the genome.

Investigating further revealed changes in normal current flow that cause real time MinKNOW read detection to split the read. Occasionally, these events trigger unblock activity, after which the read continues to sequence from the same point in the reference (in one instance this unblock loop lasted >40 minutes, then continued to sequence the same molecule, Supplementary Fig. S3). The most complex fused read observed to date consists of 38 individual reads mapping contiguously to the genome (Fig. 3d), Supplementary Fig. S4, Supplementary File Collection S2]. The plot seen in Figure 1 (Supplementary Fig. S1B) also represents a ‘fused read’. When called as a single read, the base called sequence maps contiguously to chromosome 1 from 60 882 202 to 61 129 414 bases (spanning 247 212 bases).

**Fig. 4.**
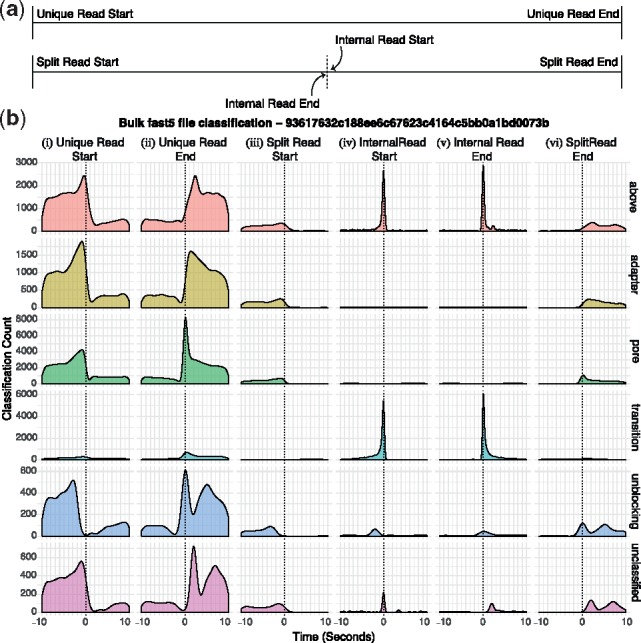
MinKNOW Classifications. Here we show selected classifications (see Supplementary Table S1 for classification definitions) captured from an entire bulk FAST5 file. (**a**) Shows the labels used for reads. Unique Read Starts and Split Read Starts are genuine new molecules being sequenced. Unique Read Ends and Split Read Ends are the real end of a read. Internal Read End and Start refers to just those incorrectly split reads. (**b**) Shows the density of each selected MinKNOW classification in a 10 second window before and after each of these read labels

Analysis of a representative bulk FAST5 file identifies annotation states correlating with the starts and ends of incorrectly split reads (Fig. 4). These are either ‘above’ or ‘transition’ classifications occuring at the change from one read to the next. At lower frequency unblocks can split reads. The ‘above’ or ‘transition’ signals can be seen in the signal traces (Fig. 2). We asked if interference from surrounding channels might cause this but grouping signals from surrounding channels failed to reveal any clear pattern (not shown).

Clearly, correcting split reads should result in more contiguous assemblies. To test this, we ran our whale_merge.py analysis across the entire data set generated by the Nanopore Human Genome consortium (Jain *et al.*, 2018). This dataset consists of 16.1 million reads with an N50 of 13 kb. Running whale_watch.py across this entire dataset identifies almost 100 000 incorrectly split reads. To demonstrate the impact of split reads on assembly we identified all reads mapping to chromosome 20 and used minimap2/miniasm to assemble reads before and after correction ( Li, 2016, 2018). Prior to correction, the assembly length was 52.5 Mb with an N50 of 3 699 497 bases. After correction, the assembly length increased to 55 Mb with an N50 of 4 673 412 bases, an N50 increase of just under 1 Mb.

## 3 Discussion

BulkVis enables visualization of bulk FAST5 files collected from Nanopore sequencers. Whilst developing BulkVis, we identified ultra-long reads can be incorrectly split by MinKNOW. This disproportionately affects ultra-long read preparations. We note that the method used for ultra-long reads is outside the normal operating conditions for nanopore sequencing (Quick, 2018). Similarly, the number of ultra-long datasets analysed in this way is limited. However, for those wishing to maximize read length the fact that adjacent reads from a single pore may represent a single molecule of DNA is significant. We have no formal explanation for why this occurs, but speculate that potential causes include DNA damage or contaminants physically linked to the DNA causing spikes in the signal. We cannot exclude the possibility that some observed split reads are caused by single strand breaks.

Additionally we note some instances where reversal of the voltage does not successfully reject a read. This effect is apparently rare and typically occurs within long reads. For applications such as selective sequencing (Loose *et al.*, 2016), reads will be rejected early in the sequencing process. We expect this will be more efficient than reads rejected midway through their length, aligning with our previous observations on ‘read until’ (Loose *et al.*, 2016). Whilst it is possible to determine the length of a read that is not rejected from a pore, it is impossible to measure the true length of reads that are successfully rejected. When running read until, reads that do not successfully unblock can be identified using the whale_watch.py script as they will appear as fused reads.

We provide helper scripts identifying candidate incorrectly split reads. These scripts are limited as they rely on suitable reference genomes to map against. It is possible to recognize candidate reads by close analysis of bulk FAST5 files although we anticipate MinKNOW itself can be further optimized to avoid incorrectly split reads. These optimizations highlight the tension between under splitting reads, leading to chimeras (White *et al.*, 2017) versus over splitting resulting in artificially shortened reads. For general use, over splitting is clearly preferential to chimeras. However for *de novo* assembly and maximizing long reads users should be aware that decisions made by MinKNOW may not be correct. In future identifying candidate incorrectly split reads from the absence of adapter sequences might be of benefit.

Whilst we see no requirement for routine collection of bulk FAST5 files, those interested in *de novo* assembly may benefit from these files. BulkVis is provided for the visual inspection of challenging or difficult to sequence samples or where the user wishes to investigate specific events during a run. In these instances analysis of a bulk FAST5 file may provide some visual indication of the underlying issues. We note that we have seen evidence of incorrect read splitting by MinKNOW across all current versions of MinKNOW and all Nanopore platforms including MinION, GridION and PromethION.

## 4 Materials and methods

### 4.1 Sequencing

Sequencing using high molecular weight DNA extracted and prepared as previously described (Jain *et al.*, 2018; Quick, 2018). RAD002 datasets are as described in Jain *et al.* (2018). RAD004 sequencing was performed using MinKNOW version 1.11.5. Standard MinKNOW running scripts were used with manual restarting to maximize the number of sequencing channels.

### 4.2 BulkVis installation and operation

BulkVis and companion scripts are available on github (https://www.github.com/LooseLab/bulkvis). Scripts make use of the python modules: NumPy (Oliphant, 2015), Pandas (McKinney, 2010), bokeh (https://bokeh.pydata.org) and h5py (Collette, 2013). Full instructions and documentation are provided at http://bulkvis.readthedocs.io.

## Supplementary Material

bty841_Supplementary_Data
