# Supplementary material for: BulkVis: a graphical viewer for Oxford nanopore bulk FAST5 files
Source: Bioinformatics. 2018 Nov 20;35(13):2193–8. doi: 10.1093/bioinformatics/bty841 (PMC6596899; doi:10.1093/bioinformatics/bty841)
Supplement: bty841_Supplementary_Data [file bty841_supplementary_data.zip › bty841-Suppl_data/Supplementary Data.pdf]

# Supplementary Materials

September 1, 2018

## Supplementary Methods

### whale_watch.py

This script takes as input a single sequencing_summmary.txt file generated by albacore or guppy (Oxford Nanopore base callers) and a paf format output file from minimap2. Optionally the user can select the distance threshold between read ends and starts for them to be considered a single molecule which defaults to 10 kb. The script outputs statistics on the input reads, the means, medians and N50s with and without correction for read splitting and a list of the top read lengths seen in an individual run. The script writes a file (default fused_reads.txt) that lists every fused read in the dataset. It provides the coordinates of the fused read in a format compatible with BulkVis to enable quick viewing.

```
python3 whale_watch.py -s /path/to/sequencing_summary.txt -p /path/to/mapping.paf
```

### whale_merge.py

This script builds on whale_watch.py to generate a FASTQ file for each detected fused read in a sequencing_summary.txt file. It requires a folder of FASTQ files in addition to the sequencing_summary.txt file and a relevant .paf file. It can either output all the reads from a run including corrected fused reads and all non-fused reads, or just output the corrected fused reads.

```
python3 whale_merge.py -s /path/to/sequencing_summary.txt -p /path/to/mapping.paf
-f /path/to/FASTQ/reads/
```

### whale_plot.py

This script is built on whale_watch.py and requires a sequencing_summary.txt, .paf file, and bulk FAST5 file to produce six CSV files containing the distributions of MinKNOW events around read starts and ends. Whale_plot.py then calls whale.R to produce a plot similar to Figure 4. Rscript and packages ggplot2, dplyr, and tidyr are required to produce the plot.

```
python3 whale_plot.py -s /path/to/sequencing_summary.txt -p /path/to/mapping.paf -b
/path/to/bulkfile.FAST5 -t 10
```

### pod_plot.py

This script generates plots for all reads in a fused_reads.txt file. This uses bokeh to render a plot (in a headless browser) and requires selenium and Pillow to be installed from pip/conda; and phantomjs from http://phantomjs.org.

```
python3 pod_plot.py -f /path/to/fused_reads.txt -b /path/to/bulkfile.FAST5 -D
output_folder
```

### bulk_info.py

Given a directory containing bulk FAST5 files this script outputs a CSV containing run information. Attributes reported are: sample frequency, run id, experiment, flowcell id, protocol version, minknow version, minion id, hostname, sequencing kit, flowcell type, asic id and experiment start time.

```
python3 bulk_info.py -d /path/to/folder/of/bulkfiles/
```

## gen_bmf.py

Given sequencing_summary.txt and mapping.paf (minimap2) files this script will convert primary mappings into coordinates that can be overlaid in BulkVis. These are saved into individual files by run ID in the output folder.

```
python3 gen_bmf.py -s /path/to/sequencing_summary.txt -p /path/to/mapping.paf
--bmf /path/to/map/folder/
```

# Supplementary Notes

## Supplementary note 1

The bulk FAST5 file used here can be downloaded from http://s3.amazonaws.com/nanopore-human-wgs/bulkfile/PLSP57501_20170308_FNFAF14035_MN16458_sequencing_run_NOTT_Hum_wh1rs2_60428.fast5

# Supplementary Tables

## Supplementary table 1

Supplementary Table 1: **Classification Descriptions.** To our knowledge there is no detailed description of the relationship between bulk FAST5 file classifications and MinKNOW labels. This table presents our assumptions about the relationship between bulk FAST5 labels and MinKNOW classifications. MinKNOW labels described on the Oxford Nanopore Forum (https://community.nanoporetech.com/support/faq/test1/minknow/minknow/what-are-the-colours-shown).

| **Bulk FAST5 file classification/s** | **MinKNOW Labels** | **Description/Comment** |
|---|---|---|
| pore, good_single, inrange | pore | A single sequencing pore is present in the channel |
| strand, strand1 | strand | DNA is detected in a single pore in the channel |
| unavailable | unavailable | A single pore which is currently blocked |
| multiple | multiple | More than one pore is detected in the channel |
| adapter | adapter | An adapter sequence is currently detected within the pore |
| mux_uncertain, unblocking | active feedback | The channel is being unblocked |
| saturated | saturated | A channel is passing too much current and has been switched off |
| zero | zero | No current is passing through the pore - likely no pore is present in the channel |
| below, user1 | out of range 1 | Negative current is being seen |
| above, user2 | out of range 2 | Current is flowing but it is neither pore nor strand |
| unclassified, unclassed | unlabelled | An unlabelled channel which has no classification assigned. |
| event | *Unknown* | No precise definition of event is available. |
| transition | *Unknown* | We believe this represents a rapid and large change in current measured. |
| unclassified_following_reset | *Unknown* | A state associated with mux changes. |
| pending_manual_reset | *Unknown* | A state associated with mux changes. |
| pending_mux_change | *Unknown* | A state associated with mux changes. |

## Supplementary table 2

Supplementary Table 2: **Read Length Statistics.** Read length statistics for 14 runs from Jain et al (Jain et al., 2018) with incorrectly split reads calculated using whale_watch.py after mapping to GRCh38 using minimap2 -x map-ont.

| Asic ID | Read count | | | Mean | | | N50 | | |
|---|---|---|---|---|---|---|---|---|---|
| | Original | Split | % | Original | Split | Corrected | Original | Corrected | Increase |
| 16056159 | 82,136 | 3,953 | 4.81 | 22,532 | 64,810 | 23,134 | 126,793 | 138,627 | 11,834 |
| 17958431 | 53,720 | 1,539 | 2.86 | 24,431 | 41,913 | 24,804 | 84,015 | 85,947 | 1,932 |
| 2901545329 | 41,384 | 932 | 2.25 | 20,299 | 51,910 | 20,534 | 59,500 | 61,168 | 1,668 |
| 3439856925 | 19,673 | 908 | 4.62 | 31,962 | 37,958 | 32,738 | 132,277 | 135,990 | 3,713 |
| 3709819546 | 73,752 | 2,489 | 3.37 | 28,268 | 56,948 | 28,777 | 129,792 | 135,156 | 5,364 |
| 3976726082 | 75,689 | 2,982 | 3.94 | 24,957 | 55,190 | 25,482 | 98,876 | 103,925 | 5,049 |
| 4109802543 | 61,223 | 2,769 | 4.52 | 26,129 | 59,149 | 26,776 | 114,934 | 123,304 | 8,370 |
| 4111860526 | 65,138 | 4,193 | 6.44 | 26,340 | 49,005 | 27,271 | 102,785 | 111,444 | 8,659 |
| 4178920553 | 270,189 | 12,045 | 4.46 | 10,680 | 14,967 | 10,936 | 26,744 | 27,759 | 1,015 |
| 4244782843 | 9,663 | 882 | 9.13 | 35,380 | 63,434 | 37,242 | 110,455 | 125,144 | 14,689 |
| 4245291640 | 72,931 | 6,860 | 9.41 | 21,243 | 55,293 | 22,410 | 102,621 | 123,768 | 21,147 |
| 4249180049 | 68,167 | 1,209 | 1.77 | 26,477 | 71,002 | 26,722 | 132,550 | 136,916 | 4,366 |
| 82266371 | 71,150 | 2,687 | 3.78 | 25,611 | 54,145 | 26,152 | 129,656 | 137,644 | 7,988 |
| 87644245 | 451,019 | 2,697 | 0.60 | 8,475 | 10,554 | 8,501 | 14,957 | 15,016 | 59 |

## Supplementary table 3

Supplementary Table 3: **Mapping data for the longest fragment.** Mapping data for eleven consecutive reads. Reads mapped to GRCh38 using minimap2 -x map-ont. The combined read length is 2,272,580 bases and maps to a total span of 2,290,436 bases.

| **Read ID** | **No** | **Ch** | **Len** | **Chr** | **Std** | **Start** | **End** |
|---|---|---|---|---|---|---|---|
| cbb741c3-27da-477e-87c8-20d91016251d | 187 | 396 | 278,221 | 12 | + | 46690621 | 46969898 |
| 7d6f6ae9-68c1-4b1e-84b5-a7aab0758c5b | 191 | 396 | 10,533 | 12 | + | 46970132 | 46979349 |
| f10eac6e-03ec-43fe-848a-769c3c85edcc | 195 | 396 | 492,326 | 12 | + | 46980918 | 47477341 |
| a19c94a3-4798-4422-a770-3122ce343de7 | 205 | 396 | 8,237 | 12 | + | 47477770 | 47485893 |
| 7c39fe08-b7d5-4160-831d-4364c7871fc4 | 206 | 396 | 231,507 | 12 | + | 47486059 | 47719483 |
| 109c8239-7357-4c20-b3a6-c1b7a95e0b48 | 208 | 396 | 199,576 | 12 | + | 47719572 | 47920652 |
| 0e63d0f4-0e41-4eb8-bfd2-69143efb70f5 | 211 | 396 | 148,542 | 12 | + | 47920996 | 48071262 |
| 760d1f30-ac17-4026-84e5-a94856c6d650 | 212 | 396 | 24,966 | 12 | + | 48071616 | 48096201 |
| ac191a38-ce73-4610-ae5b-8fbe03178ad7 | 216 | 396 | 542,458 | 12 | + | 48096764 | 48640714 |
| 07df9239-2302-436c-9430-048c6061c848 | 223 | 396 | 216,645 | 12 | + | 48640869 | 48859917 |
| c371771b-016c-4bfc-9dae-592e6631d0ba | 231 | 396 | 119,569 | 12 | + | 48860239 | 48981057 |

## Supplementary table 4

Supplementary Table 4: **Longest read with a bulkfile** Mapping data for nine consecutive reads. Reads mapped to GRCh38 using minimap2 -x map-ont. The combined read length is 1,385,925 bases and maps to a total span of 1,470,878 bases.

| Read ID | No. | Ch | Len | Chr | Std | Start | End |
|---|---|---|---|---|---|---|---|
| 3ce0651d-5a64-4d57-bd5c-3cd57045d473 | 325 | 56 | 262,945 | 13 | - | 46000683 | 46279489 |
| 6588b110-ef2c-48a2-b722-74902345f0dd | 329 | 56 | 53,061 | 13 | - | 45944001 | 46000240 |
| 5e3eeb11-9f03-45c5-b00e-99bdab5c1c3d | 333 | 56 | 21,821 | 13 | - | 45921320 | 45943803 |
| 07a93f40-2e33-451d-bb00-623d5bdebdfd | 338 | 56 | 3,096 | 13 | - | 45917140 | 45920055 |
| 1fac5b9f-9a73-45e4-a8d1-5336161f08c6 | 339 | 56 | 164,502 | 13 | - | 45742838 | 45916806 |
| c6a7d4b5-252b-4665-bc1e-77a315154edc | 340 | 56 | 300,951 | 13 | - | 45424411 | 45742140 |
| c0d044e3-668d-4794-a535-c77a16fb850a | 344 | 56 | 94,514 | 13 | - | 45323669 | 45423042 |
| 491016a6-b817-4af9-8fd5-9a3572326c95 | 348 | 56 | 370,342 | 13 | - | 44930258 | 45320732 |
| 8ed6cd0a-f4e6-4785-810d-f8193688bdf0 | 350 | 56 | 114,693 | 13 | - | 44808611 | 44929104 |

# Supplementary Figures

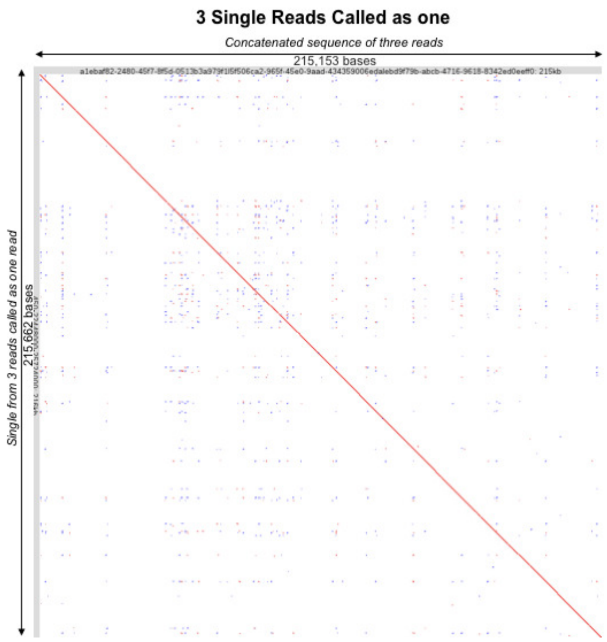


(a)

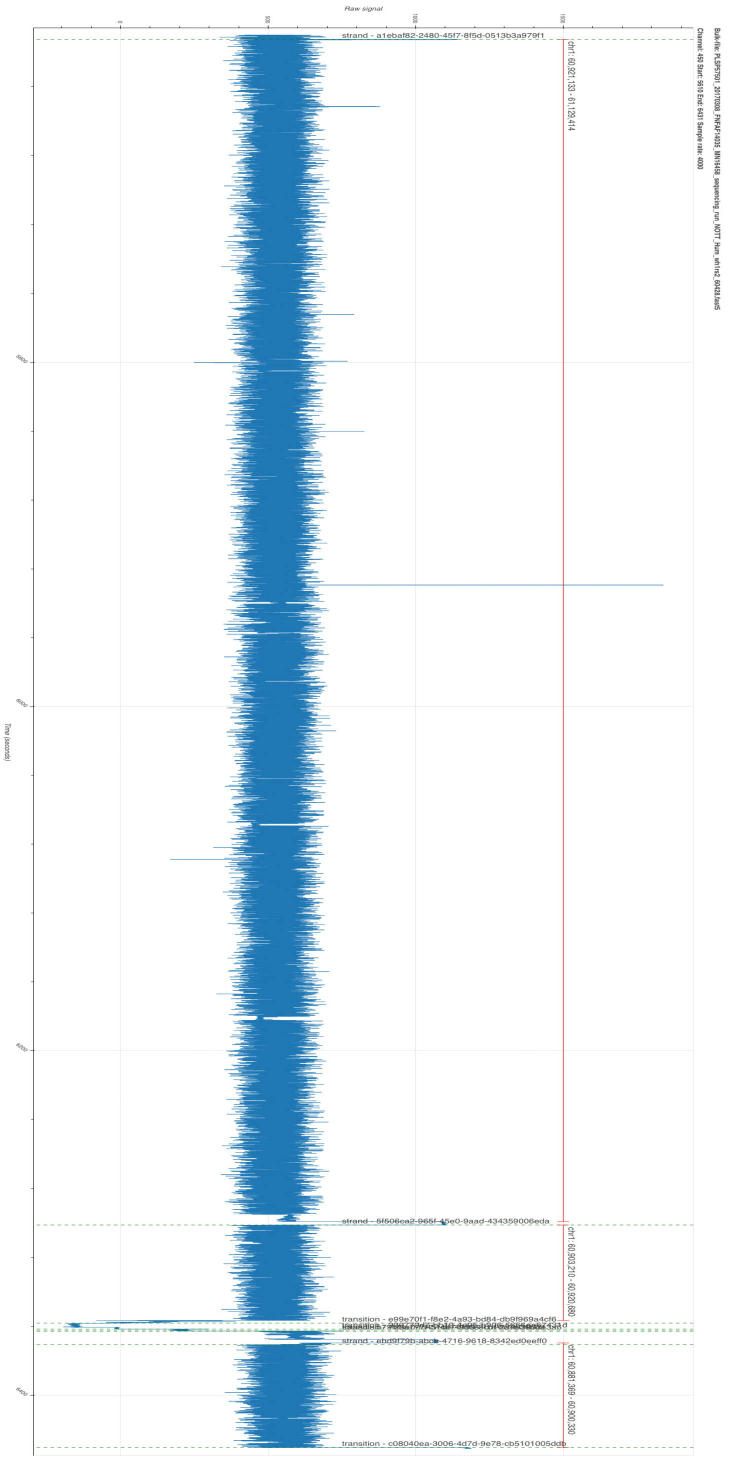

(b)

Supplementary Figure 1: (**a**) simple last alignment and dot plot of the three individual basecalled reads shown in Figure 1 aligned against the merged signal for each of those three reads called as one read by BulkVis. (**b**) Signal of reads shown in Figure 1 with mappings overlaid.

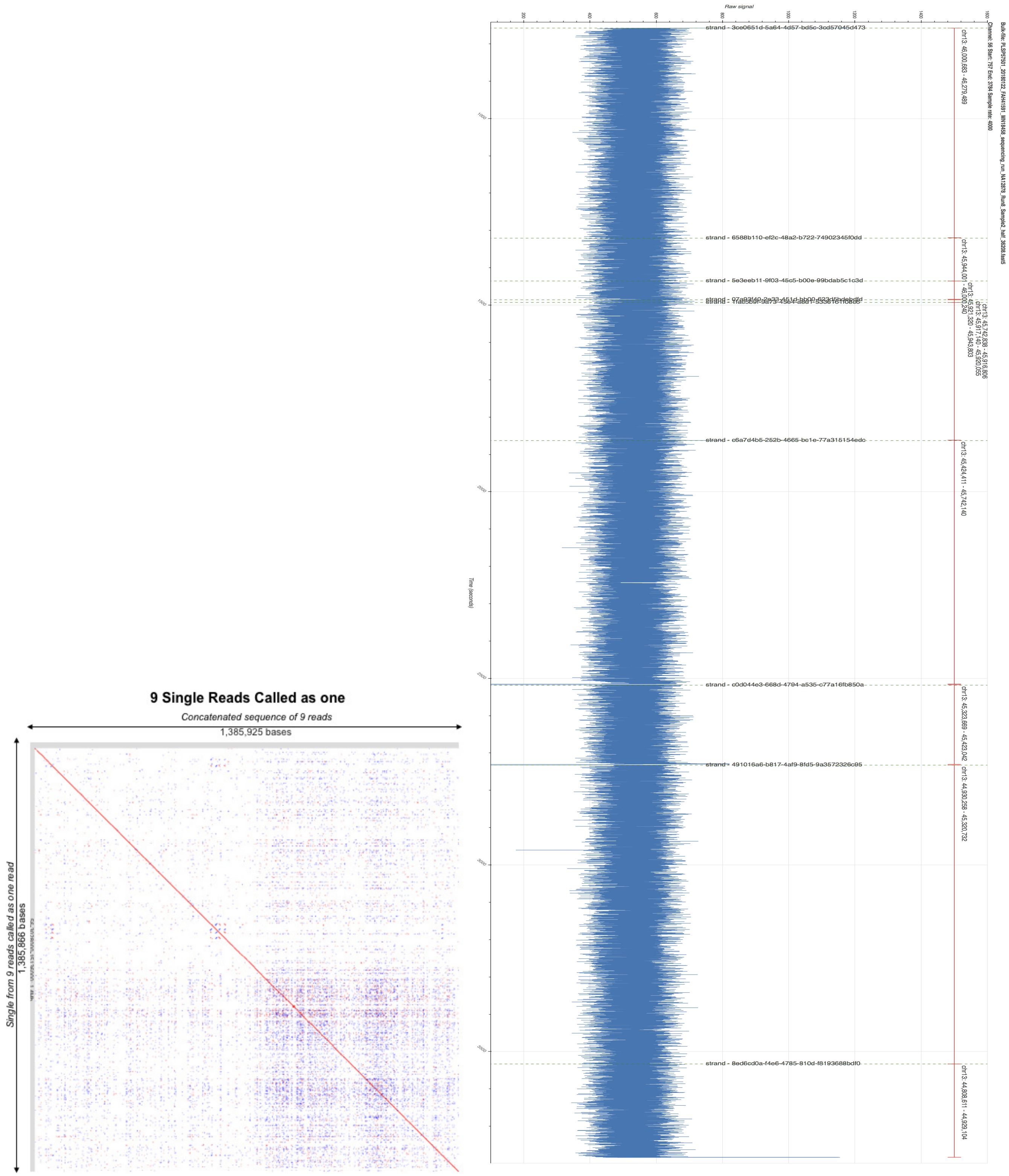


(a) (b)

Supplementary Figure 2: (**a**) Mapping the concatenated basecalled reads against the single read called from squiggle by BulkVis. (**b**) The full length signal region spanning 9 individual reads from a bulk FAST5 file as shown in Figure 3c. Dashed lines indicate new reads identified by MinKNOW. Mappings are overlaid above.

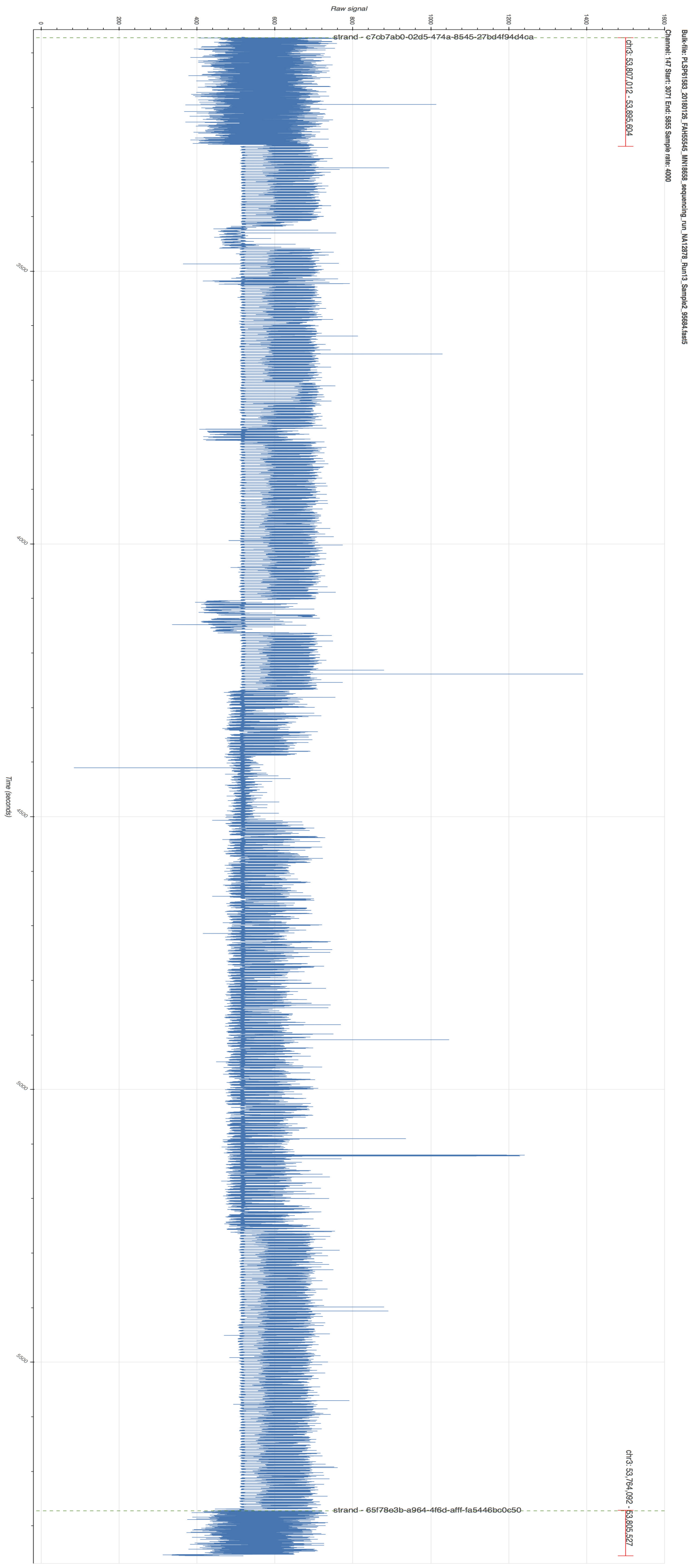


Supplementary Figure 3: A read containing an unblock sequence which lasts over 46 minutes, but later continues to sequence the same molecule. Dashed lines indicate new reads identified by MinKNOW. The boxed region contains repeated unblocks.

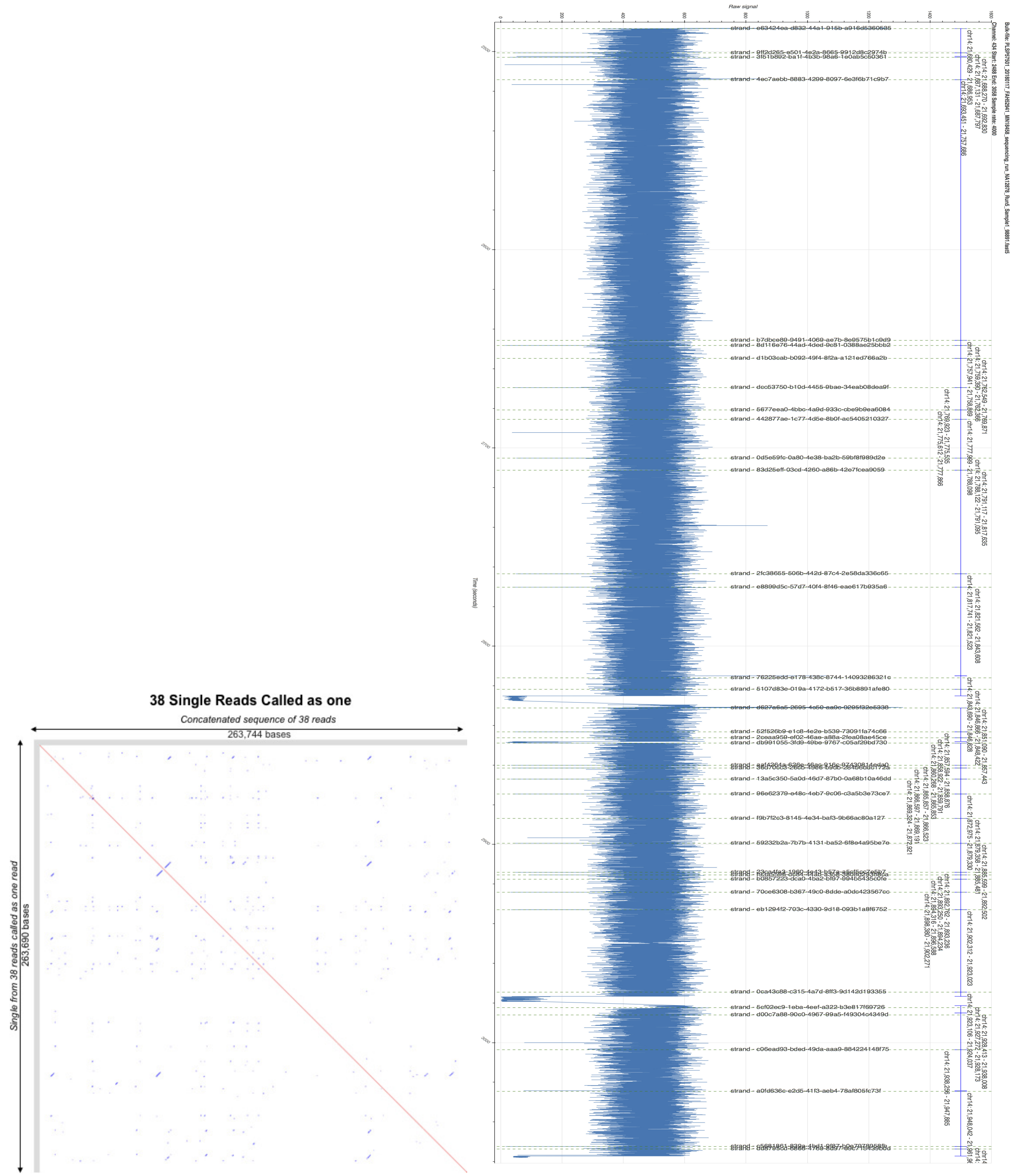


(a) (b)

Supplementary Figure 4: (**a**) Mapping the concatenated basecalled reads against the single read called from squiggle by BulkVis. (**b**) The full length signal region spanning 38 individual reads from a bulk FAST5 file as shown in Figure 3d. Dashed lines indicate new reads identified by MinKNOW.

# Supplementary Files

## Supplementary File Collection1

This file is a tar.gz archive containing the sequencing_summary.txt and mapping.paf from the MinION with ASIC ID 3976726082. The ‘bulkvis’ folder contains a read FAST5 file generated by BulkVis and two FASTQ files that were created by calling the BulkVis read FAST5 file and merging the original split FASTQ files; the ‘original’ folder contains the read FAST5 files as originally split by MinKNOW and their resulting FASTQ file.

## Supplementary File Collection2

This file is a tar.gz archive containing four subfolders: 3A_longest-single-read, 3B_longest-fused-read-without-bulkfile, 3C_longest-fused-read-with-bulkfile, and 3D_read-with-38-splits; each of these folders holds an ‘original’, ‘bulkvis’, or both folders. The ‘original’ folders contains the read FAST5 files as originally split by MinKNOW and their resulting FASTQ files; the ‘bulkvis’ folders contains read FAST5 files generated by BulkVis and FASTQ files that were created by calling the BulkVis read FAST5 file and merging the original split FASTQ files.
